# Carbon Sink Cost and Influence Factors Analysis in a National Afforestation Project under Different Investment Modes

**DOI:** 10.3390/ijerph19137738

**Published:** 2022-06-24

**Authors:** Xuexiang Li, Wanlin Hu, Fan Zhang, Jinxin Zhang, Feng Sheng, Xiangyu Xu

**Affiliations:** 1 School of Business, Hubei University, Wuhan 430062, China; 202011112010082@stu.hubu.edu.cn; 2 Research Center for China Agriculture Carbon Emission Reduction and Carbon Trading, Hubei University, Wuhan 430062, China; 20170022@hubu.edu.cn; 3 College of Economics & Management, Huazhong Agricultural University, Wuhan 430070, China; huwanling0816@163.com; 4 School of Soil and Water Conservation, Beijing Forestry University, Beijing 100091, China; 5 State Key Laboratory of Biocatalysis & Enzyme Engineering, College of Life Science, Hubei University, Wuhan 430062, China; 6 Institute of Plant Protection and Soil Fertilizer, Hubei Academy of Agricultural Sciences, Wuhan 430064, China; xuxy@hbaas.com

**Keywords:** carbon sink cost, national afforestation project, impact factors, direct investment, public–private partnership investment, Laohekou city

## Abstract

Afforestation projects are the main source of carbon sink. Measurement and impact analysis of carbon sink costs will help accelerate the marketization of forestry carbon sink. Considering the opportunity cost of land use and the carbon release cost of wood products, this study proposed a forestry carbon sink cost model under the Public–Private Partnership (PPP) and the direct (DI) investment mode based on the classic carbon sink model. Then, the proposed models were applied to a real-world afforestation project, the 20-year national afforestation project (NAP) in Laohekou City, Hubei Province, China. With the help of the input–output forestry carbon sink cost–benefit analysis framework, the dynamic analysis of factors such as rotation period, timber price, discount rate and yield rate for forestry is carried out. Results show that: (1) with the increasing of rotation period, wood market price, and wood yield rate, the carbon sink cost of Laohekou NAP gradually decreases, while the discount rate has the opposite trend; (2) the DI mode is more feasible than the PPP model at the present condition. The PPP mode is more feasible than the DI mode only when the wood price is lower than 73.18% of the current price, the yield rate is lower than 0.485, and the discount rate is higher than 6.77%. (3) When choosing tree species for NAP, the carbon sink capacity, wood market price, maturity time, and planting cost should be synthetically considered. The proposed model and the obtained results can not only support local governments and forestry carbon sink enterprises to make tradeoffs between PPP and DI mode, but also provide them with useful information for reducing carbon sink costs.

## 1. Introduction

As one of the salient features of climate change, global warming has many adverse impacts on human welfare and social development [1]. Many countries have proposed specific measures to mitigate global warming and reduce greenhouse gas emissions. China is the largest emitter of carbon dioxide around the world [2]. According to the statement of President Xi Jinping at the general debate of the 75th session of the United Nations General Assembly, China would scale up its Nationally Determined Contributions (NDCs) by adopting more vigorous policies and measures, and aims to have CO_2_ emissions peak before 2030 and achieve carbon neutrality before 2060 [3,4]. The current measures to reduce greenhouse gas emissions can be roughly divided into two categories: one is direct emission reduction, which mainly refers to restricting carbon emissions from energy-consuming industries; the other is indirect emission reduction via increasing carbon absorption and improve carbon sink capacity of forests, grasslands, wetlands, oceans, soil, and other ecosystems. As the largest ecosystem on land, forests play an irreplaceable role in coping with climate change. Studies have indicated that forest trees generally absorb 1.83 tons of carbon dioxide and release 1.62 tons of oxygen per cubic meter of growth [5]. Compared with reducing greenhouse gas emissions, forest carbon sinks have obvious cost advantages [6]. At present, forests are regarded as not only an important ecosystem for our human beings, but also the major carbon sink pathway for mitigating climate change.

Afforestation is always regarded as an important pathway achieving carbon neutrality. A forest is an economic, social, and environmental asset because it can generate economic value, produce non-timber products, fix carbon dioxide, and reduce carbon dioxide emissions by replacing fossil fuels. The development of forestry can not only bring economic income to areas rich in forest resources, but also realize its economic value on the platform of carbon trading market through its contribution to climate change. Although forestry carbon sinks have been mentioned many times, how to develop and utilize them is still a new topic [7]. In addition, China has only 5% of the world’s forest area and 3% of the world’s forest stock while supporting the demand for ecological and forest products of 23% of the world’s population [8]. Carbon neutrality goal and intense contradiction between supply and demand jointly request expending the forest area and increasing forest stock for sustainable development in the future. In order to ensure the wood safety and alleviate the structural shortage of rare tree species and general tree species with large-diameter grades, China has started to conduct a national afforestation project (NAP). NAP aimed to foster multi-functional forests (such as wood production, carbon sink, and ecosystem protection) in suitable areas with good planting conditions and will supply subsidies to corresponding enterprises and farms that undertake forest tending tasks. According to related planning report of China [9], over 2.0 × 10^7^ ha NAP will be planted by 2035. These forests will absorb 300 million tons of carbon dioxide, accounting for about 30% of China’s forest carbon sink system. NAP is a very important forest carbon sink pathway and plays a very important role in China’s carbon neutralization, and thus the Chinese government has provided some fiscal policies to promote NAP construction. For example, they always give construction cost subsidy, tax deduction, loan priority, and loan rate discount for NAP-participating businesses.

In the carbon trading market, forest carbon sinks can be traded as common commodities, thereby helping some countries and enterprises to ease the pressure on emission reduction. Commodity trading is inseparable from prices, and the price mechanism is the core of the market mechanism. The current carbon sink price of China is seriously deviated from the value, which affects the supply capacity of the forest carbon sink system and the enthusiasm of market players to invest in NAPs. Therefore, it is necessary to scientifically measure the carbon sink cost of NAP and identify low-cost scenarios, which have important practical and policy value for promoting the development and investment in NAP and helping government department formulate specific actions to address climate change. Generally, there are two typical investment mode in NAP, including Public–Private Partnership (PPP) and direct investment (DI) mode [10]. In PPP mode, the government will provide some policy subsidy, and withdraw the NAP from the enterprise after project execution period. That is, wood will not be allowed to be felled for selling. For DI mode, the enterprises will independently invest in afforestation and sell wood. These two modes obviously affect the carbon sink cost of NAP, and their impacts need to be further discussed.

Many efforts have been made for calculating carbon sink cost [11], and these methods can be divided into two types. One type is to calculating carbon sink cost from a cost perspective, including afforestation cost method [12], artificial fixed CO_2_ cost method, and carbon tax method [13]. The other type is to calculating carbon sink cost from a market benefit perspective, such as market value method [14], cost–benefit method, and payment willingness method [15]. Due to incomplete carbon sink trading and no carbon tax, the forestry carbon sink value in China is always approximated by the afforestation cost method [16]. Afforestation cost methods have been widely used for calculating the carbon sink cost of forest. For example, SoEun Ahn [17] ran an econometric model with land use and provincial panel data and calculated the unit carbon sink cost of forest in South Korea. Guo et al. [18] used a partial equilibrium model to estimate the impacts of carbon price on timber harvest volume and price in different time periods and on the change of forest carbon stock over time in Sweden. Cao et al. [6] developed an economic carbon sink cost measurement model for the *Pinus elliottii* afforestation project. These studies estimated the forest carbon sink cost and analyzed the impacts of key factors based on the proposed model. However, few studies paid attention to the influence of different investment modes on the carbon sink cost in practical afforestation project, which should be further discussed to help governments and enterprises run NAP.

Therefore, considering the opportunity cost of land use and the carbon release cost of wood products, this study proposed a forestry carbon sink cost model under the PPP and the DI investment mode based on the classic carbon sink model [19]. Then, the proposed models were applied to a real-world afforestation project, the 20-year national afforestation project in Laohekou City, Hubei Province, China. With the help of the input–output forestry carbon sink cost–benefit analysis framework, the dynamic analysis of factors such as rotation period, timber price, discount rate, and yield rate for forestry was carried out. The proposed model and the obtained results can not only provide decision support for local governments and forestry carbon sink enterprises, but also provide decision-making tools for forestry carbon sink planning in similar regions.

## 2. Model Formulation

This study attempted to evaluate the impacts of carbon sink via modeling approach. The woodland net present value model and woodland carbon sink cost model under different investment modes will be detailed and introduced in this section, which will be used to measure the carbon sink cost of NAP. The scope of this study is afforestation projects that use barren hills and wasteland, which are regarded as potential agricultural lands to plant crops. This model can be used in scenarios in which governments and enterprises have to choose an investment mode and estimate carbon sink cost.

### 2.1. Modified Hartman Model for Woodland Net Present Value Calculation

The woodland net present value (*NPV*) can be expressed as the sum of the net present value of wood value and non-timber value under the condition of infinite rotation period [20]. Non-timber value mainly comes from the recreational value of forests, water conservation, carbon sink services, biodiversity, and some non-timber products, such as dried and fresh fruits. In this study, only the carbon sink value is considered. The net present value of forest land is closely related to the rotation period. When the wood is in the best rotation period, the value is the largest, and the net present value of the woodland is called the woodland price. Therefore, the revised Hartman model under the condition of infinite rotation period, the *NPV* model expression of forest land, is [21]:

(1) $$NPV^{\infty }=NPV_{t}+NPV_{c}=\frac{P_{t}Q\left(t\right)e^{-rt}+\int_{0}^{t}P_{c}Q^{\prime }\left(t\right)e^{-rt}dt-C_{f}-C_{m}-C_{h}}{1-e^{-rt}}$$

where $NPV^{\infty }$, *NPV_t_*, and *NPV_c_* are net present value of forest land under infinite rotation, net present value of timber income, and net present value of carbon sink income, respectively; *P_t_* and *P_c_* are the wood price and carbon price, respectively; *Q*(*t*) is the stock volume per unit area when the tree age is *t*, which can be obtained from the accumulation growth model or query volume table; *Q*′(*t*) is the derivative of *Q*(*t*) that represents the annual accumulation growth during *t* th year; *r* is the continuous discount rate, and the discount rate is equal to bank’s long-term deposit interest rate in this study; *C_f_*, *C_m_*, and *C_h_* respectively represent the cost of initial planting cost, annual management cost, and harvesting transportation cost. Taking the derivative of Equation (1) with respect to tree age *t* and making it equal to zero, the maximum *NPV* of forest land under the optimal rotation period can be obtained.

Assuming that the carbon sink project is afforestation on bare land, the same afforestation cost will be paid after a rotation period, and no logging will be carried out during the rotation period. Part of the cost needs to be discounted, and the yield rate δ of wood is considered at the same time. Then, the net present value of timber income is:

(2) $$NPV_{t}=\frac{\delta \left(P_{t}-C_{h}\right)Q\left(t\right)e^{-rt}-C_{f}-C_{m}}{1-e^{-rt}}$$

Besides, because the data of underground carbon pool and ground litter carbon pool are difficult to obtain, only above-ground biomass carbon pool is considered in this paper. When considering the carbon sink value, it is necessary to convert the aboveground trunk stock into carbon content and consider the carbon release cost of wood products. Therefore, the net present value model of carbon sink income is:

(3) $$NPV_{c}=\frac{\int_{0}^{t}\lambda P_{c}Q^{\prime }\left(t\right)e^{-rt}dt-P_{c}\left(1-\alpha \right)R\cdot e^{-rt}}{1-e^{-rt}}$$

where *λ* is the carbon conversion coefficient, which is obtained from the product of basic wood density (*D*), biomass expansion factor (*BEF*), aboveground biomass percentage (1-*θ*) and biomass carbon content (*CF*), according to the Methodology of Carbon Sink Forestation Project [22]; *R* denotes the carbon content per unit area (ha) of the forest tree when it is harvested at the end of a rotation period, which can be calculated through multiplying the unit volume *Q(t)* and the carbon conversion coefficient *λ*; *α* represents the proportion of carbon fixed in wood products for a long time (when *α* = 0, the carbon in all wood products will be released into the atmosphere through combustion or other means; when *α* = 1, the carbon of the harvested wood is all stored in the wood for a long time). In this study, α is regarded as a function of the decay rate of wood products, and the carbon release rate caused by decay of wood products is an exponential equation of time *t* [23]. Next, the carbon content *W(t)* in wood products can be calculated as:

(4) $$W\left(t\right)=\beta \cdot R\cdot e^{-V_{1}\left(t-T\right)}+\left(1-\beta \right)\cdot R\cdot e^{-V_{2}\left(t-T\right)}$$

where *T* is the rotation period, and (*t* − *T*) is the time after felling; *V*_1_ is the decay rate of long-term durable wood products (according to the research of Sohngen and Sedjo [23], *V*_1_ is 0.79% per year, and *V*_2_ is the decay rate of short-term wood products per year, which is 1.03%); *β* is the proportion of long-term durable goods in wood products, which is 50% in this study. Through calculating the derivative of the tree age *t* in Formula (4), *W*’(*t*), the carbon release rate of wood products can be obtained as follows:

(5) $$W^{\prime }\left(t\right)=-\beta \cdot R\cdot V_{1}\cdot e^{-V_{1}\left(t-T\right)}-\left(1-\beta \right)\cdot R\cdot V_{2}\cdot e^{-V_{2}\left(t-T\right)}$$

Assuming that the carbon content *R* of the wood is only stored in the following two parts of wood products (one part is in long-term durable solid wood products, such as building materials and furniture, this part of the carbon release rate is slow; the other part is in short-term products, such as packaging materials, this part of carbon is released fast), the net present value of carbon sink benefits in one rotation period is expressed as:

(6) $$NPV_{c}=P_{c}\cdot R\cdot e^{-rT}-P_{c}\int_{t=T}^{\infty }W^{\prime }\left(t\right)e^{-rT}dt$$

Through adding Formula (5) into Formula (6), the following formula can be obtained:

(7) $$NPV_{c}=\left[1-\frac{V_{1}\cdot \beta }{V_{1}+r}-\frac{V_{2}\cdot \left(1-\beta \right)}{V_{2}+r}\right]\cdot P_{c}\cdot R\cdot e^{-rt}$$

With the help of Formula (3), the net present value of carbon sink income in one rotation period can be generated as:

(8) $$NPV_{c}=\alpha \cdot P_{c}\cdot R\cdot e^{-rt}$$

According to the Formulas (7) and (8), the proportion of carbon fixed in wood products for a long time is:

(9) $$\alpha =1-\frac{V_{1}\cdot \beta }{V_{1}+r}-\frac{V_{2}\cdot \left(1-\beta \right)}{V_{2}+r}$$

### 2.2. Woodland Carbon Sink Cost under Different Investment Modes

It is assumed that land managers can autonomously choose two ways of land use, including agricultural production and forestry carbon sink production. If the land in area is used for agricultural production and the crop production cycle is one year, the net present value of the agricultural income per unit area (ha) of the land within 1 year is:

(10) $$n_{i}=Q_{i}-C_{i}$$

where *i* is the index of region; *Q_i_* denotes the gross agricultural production value per unit area in one year (excluding forest, animal husbandry, and fishery); *C_i_* is the agricultural planting cost per unit area in one year. Then, the sales profit margin *P_i_* of agricultural production is:

(11) $$P_{i}=\frac{n_{i}}{Q_{i}}\times 100\%$$

Assuming that the planting cost and income per unit area are unchanged, the net present value of agricultural land in an infinite production period *A_i_* is [24]:

(12) $$A_{i}=n_{i}\cdot \sum_{i=1}{\left(1+r\right)}^{\left(1-t\right)}=\frac{n_{i}}{r}$$

In this study, the annual agricultural income profit rate *P_i_* per unit area and the discount rate *r* are assumed to be equal. According to the Formulas (11) and (12), the following equation can be obtained:

(13) $$A_{i}=Q_{i}$$

The above equation indicates that the net present value of the permanent income per unit area for agricultural production is equal to its annual gross agricultural output value (excluding forestry, animal husbandry, and fishery).

When land owners make a choice between agricultural production and carbon sink production, they are more inclined to land use strategies with higher net present benefits according to theory of ‘rational economic person’. Therefore, only if the net present value of the permanent benefits used to produce carbon sinks is higher than agricultural production, the land owners will engage in carbon sink production. In other words, forestry carbon sink price can reach the lowest point when the production carbon sink is equal to the net present value of the permanent benefits of agricultural production. The lowest carbon sink cost under PPP and DI mode can be respectively expressed as Formulas (14) and (15).

(14) $$P_{c}=\left(1-\omega \right)\frac{A\left[1-e^{-\left(r-s\right)t}\right]+C_{f}+C_{m}}{\int_{0}^{t}\lambda \cdot Q^{\prime }\left(t\right)\cdot e^{-\left(r-s\right)t}dt}\times \frac{12}{44}$$

(15) $$P_{c}=\frac{A\left(1-e^{-rt}\right)-\left[\delta \cdot \left(P_{t}-C_{h}\right)\cdot Q\left(t\right)\cdot e^{-rt}-C_{f}-C_{m}\right]}{\int_{0}^{t}\lambda \cdot Q^{\prime }\left(t\right)\cdot e^{-rt}dt-\lambda \cdot \left(1-\alpha \right)\cdot Q\left(t\right)\cdot e^{-rt}}\times \frac{12}{44}$$

where *s* and *ω* indicate the subsidy discount rate and cost supported by the government. The calculation result of the above formulas is unit carbon sink price. The main differences of PPP mode are: (1) companies cannot sell wood for profit, so wood sales revenue *P_t_* and carbon loss are not considered; (2) the government will subsidize the discount rate and cost, which will reduce investment cost of enterprises. After obtaining the carbon sink cost of different tree species and the weighted average according to their different planting areas, the total carbon sink cost of the project can be obtained.

Generally, carbon sink production only occurs in areas where the market carbon sink price is higher than the carbon sink cost. The lower the carbon sink cost, the higher the carbon sink income.

## 3. Case Study

### 3.1. Overview of Study Area

**Figure 1:**
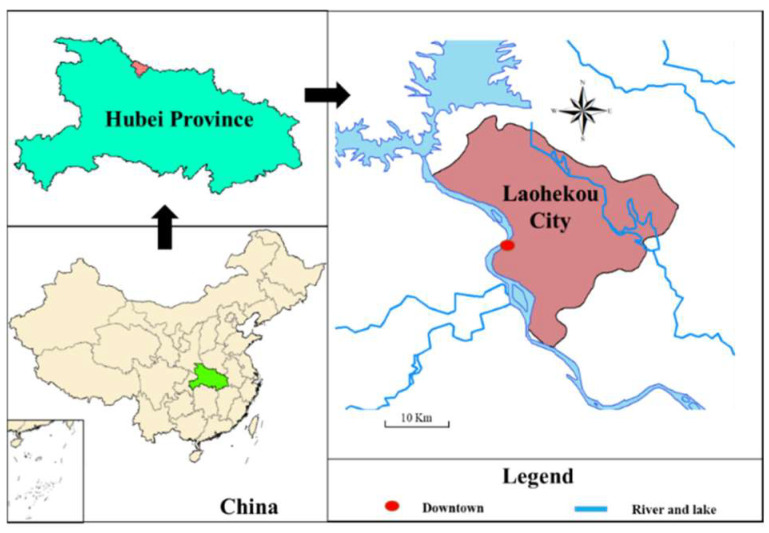
Geolocation of the study area.

The study area is the national afforestation project in Laohekou City (111°30′–112°00′ E, 32°10′–32°38′ N) (Figure 1), Hubei province, in China. Hubei is in the central part of China. In order to strengthen ecological construction, Hubei Province has implemented the natural forest protection project, the Yangtze River shelter forest project, the project of returning farmland to forest, and the comprehensive control project of rocky desertification. Through analyzing the wood demand of wood processing enterprises in Hubei Province during past ten years, we found that the supply and demand ratio of the wood market has fluctuated around 50% and 65%, respectively. With the development of the paper production industry and the wood-based panel manufacturing industry, the wood supply gap is more than 2 million cubic meters, and there is an obvious contradiction between supply and demand. From 2011 to 2015, the annual timber yield in the Hubei province was about 5 million cubic meters, and the shortage of timber was nearly 7 million cubic meters, which has reached more than 8 million cubic meters during 2016–2020. Due to the shortage of large-diameter timber and rare tree species resources in Hubei Province, production enterprises that use timber as raw materials can only rely on medium and small-diameter timber and non-rare tree species, leading to a low quality of processed forest products and a low utilization rate of wood resources.

Laohekou City is in the northwest of Hubei Province. This area is mainly covered by yellow-brown loam, accounting for 67.7%. The pH value of soil is 5.83–7.08, which is slightly acidic to slightly alkaline and very suitable for the growth of various forest plants. According to the related Survey Report in Hubei Province [25], the forestry land area of Laohekou City is 18,322.81 ha, including 12,156.97 ha of arbor forest, 88.39 ha of bamboo forest, 62.30 ha of sparse forest, 3617.06 ha of shrub forest, undeveloped forest land is 678.32 ha, the nursery land is 146.13 ha, the forest land without trees is 209.30 ha, and the suitable forest land is 1343.74 ha. The city’s forest coverage rate is 13.79%, and the forest greening rate is 17.62%. The city’s living wood volume is 464,700 m^3^, of which the forest volume is 326,400 m^3^, and the scattered wood volume is 4800 m^3^.

To increase forestry carbon sink and wood stock, the government of Laohekou City have planned to construct the NAP since 2021. By creating high-efficiency plantations and cultivating large-diameter native tree species, the project can increase the timber storage and carbon sinks in Laohekou City, which can promote the sustainable development of wood industry and contribute to the carbon neutral. According to the field survey and local reports, the grain yield and population of Laohekou city is around 350.93 million kg and 420.00 thousand people. That is, every person in Laohekou city can be offered around 835.55 kg per year, which is twice as much as the international food safety standard line (400.00 kg per year). The lands used in the Laohekou NAP are barren mountains and virgin lands. The climatic conditions of Laohekou are particularly suitable for planting plants, including grain crops and trees. These lands are usually considered as potential agricultural land resources, and thus it is necessary to consider the opportunity cost of the land. Trees used in this NAP construction can be divided into five types, including Precious deciduous hardwood species (such as *Acer buergerianum Miq*., *Catalpa bungei C. A. Mey*, *Ulmus pumila Linn*., and et.), coniferous species (such as *Metasequoia*, *Taxodium ascendens Brongn*, *Pinus elliottii Engelmann*., and et.), native hardwood species (such as *Bischofia polycarpa (Levl.) Airy Shaw*, *Melia azedarace L.*, *Sophora japonica L*., and et.), fast-growing broadleaf tree species (mainly refer to *Paulowinia*.), and Oil tree species (mainly refer to *Idesia polycarpa Maxim.*). Some related data will be introduced in the following section.

### 3.2. Data Preparation

**Figure 2:**
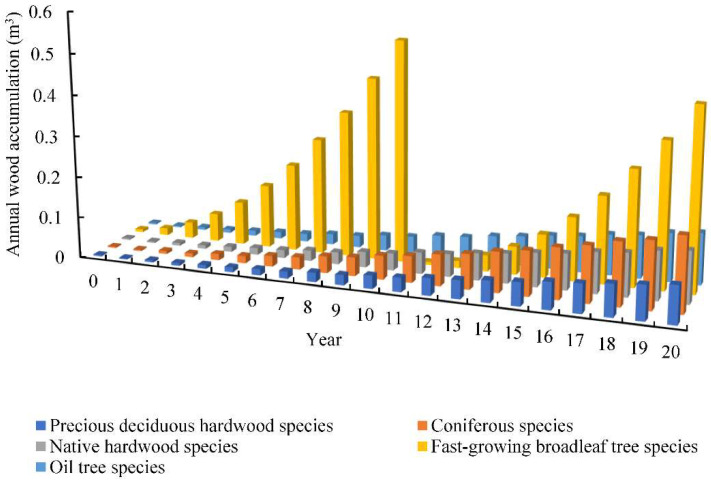
Annual wood accumulation of five tree species in Laohekou NAP.

The necessary data of Laohekou NAP will be collected in this section. According to the Xiangyang Statistical Yearbook (2021) [26], the annual gross agricultural output value (excluding forestry, animal husbandry, and fishery) and land for agricultural production are 7.66 × 10^8^ USD and 9.44 × 10^4^ ha, respectively. Thus, the net present value of agricultural land can be obtained as 8.12 × 10^3^ USD/ha. The bank rate is 4.90%, and the government subsidies rate in PPP mode is 3%. Furthermore, we found that government subsidies 30% of the total cost of the investment in Laohekou city. The NAP implementation period is 20 years in this study. Besides, annual wood accumulation of five tree species in Laohekou NAP can be found in the book of Chinese vertical timber volume table [27] (Figure 2). Other detailed data of five tree species in Laohekou NAP were collected from the field survey, regional report, and literature review, which are displayed in Table 1.

## 4. Results Analysis

### 4.1. Carbon Sink Cost with Different Rotation Periods

**Figure 3:**
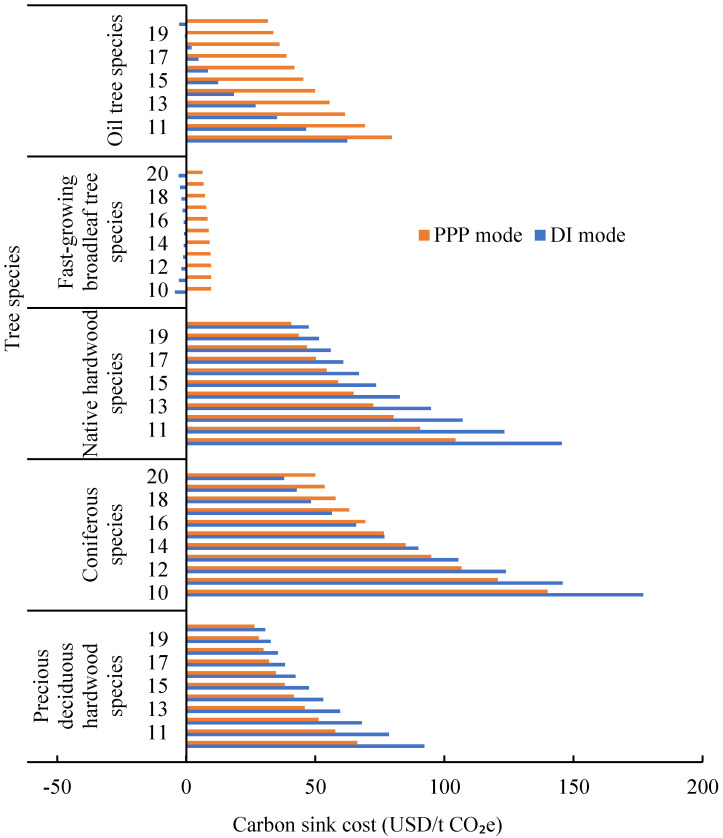
CO_2_ sink cost of different tree species in Laohekou NAP.

After inputting the data to Formulas (14) and (15), the CO_2_ sink cost of different tree species in Laohekou NAP during 10–20 rotation period under the DI and PPP mode can be generated as Figure 3 shows. With the increase of the rotation period, there is a decreasing trend in the CO_2_ sink cost of almost tree species under two investment mode, except fast-growing broadleaf tree species with DI investment mode. It changes from −4.39 USD/t CO_2_e in 2030 to −0.81 USD/t CO_2_e in 2035, and finally to −2.87 USD/t CO_2_e in 2040. The main reason is that these species have a fast growth rate which has two rotation periods within 20 years, and have the highest carbon sink capacity among five tree species. Additionally, it can be found that the cost advantage has changed in Coniferous species. When the rotation period is less than 14 years, the CO_2_ carbon sink cost under the DI mode is higher than PPP mode, and when the rotation period exceeds 14 years, the DI mode will have more advantages than the PPP mode. For Precious deciduous hard-wood and native hardwood species, DI has an obvious advantage in every rotation period. Among the five tree species, Oil tree species has the highest planting density and lowest planting cost, in which the DI mode has absolute advantages compared with the PPP mode. Such information can give governments some implications for conducting NAP programs as: (1) when the tree species in the project are mainly fast-growing broadleaf trees and Oil tree species, the PPP investment mode may not suitable for NAP construction; (2) When the implementation period of the NAP is short and the tree species are mainly coniferous, Precious deciduous hard-wood, and native hardwood species, the PPP mode can reduce the cost of carbon sinks and effectively attract commercial investment compared with the DI. (3) Whether it is the direct investment model or the PPP model, the longer the rotation period, the lower the cost.

**Figure 4:**
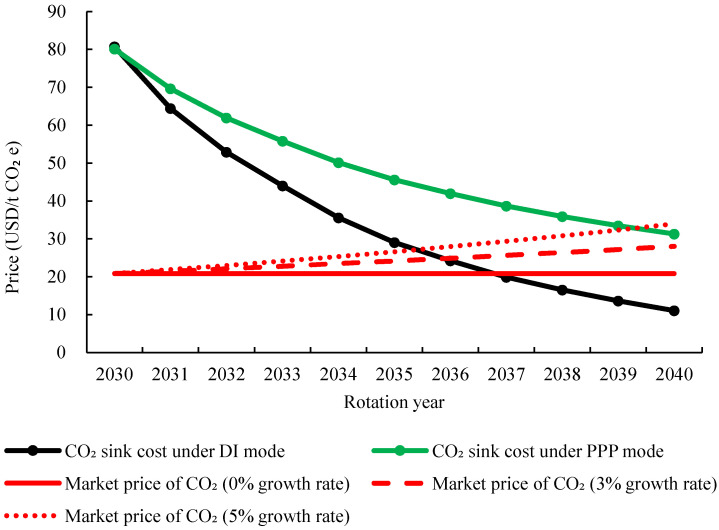
Sink cost and future market price of CO_2_ in Laohekou NAP.

Besides, this study simulates and calculates the changes of total carbon sink cost of Laohekou NAP. The calculation results can be found in Figure 4, indicating that with the increase of the rotation period, the CO_2_ sink cost of Laohekou NAP will decrease. The CO_2_ sink cost at the end of the Laohekou NAP under DI and PPP mode is 11.06 and 40.94 USD/t CO_2_e. According to the China Carbon Price Survey Report (2021) [28], the average carbon price in the national carbon market in 2022 will be 7.35 USD/t CO_2_e, and it will rise to 13.06 USD/t CO_2_e by 2025 with an average annual increase of more than 20%. It may further reach 20.86 USD/t CO_2_e before 2030. Assuming annual carbon market-price growth rates of 0%, 3%, and 5%, the average market price during 2030 to 2040 can be predicted as Figure 4 shows. It is noteworthy that the DI mode can gain profit around 2036 under all three growth rates, while PPP mode is only profitable at the end of the project if the carbon market price growth rate is 5%. That is, existing subsidy measures are not enough to motivate enterprises to choose the PPP model. If the government wants to implement the PPP mode in Laohekou NAP, some cost reduction measures, such as extending the benefit period of enterprises, should be considered. Besides, NAPs have multiple sociological implications, including increasing biodiversity, mitigating soil erosion, providing habitats for human and animals, and generating income for farmers. If these sociological implications can be measured in the cost calculation and bring benefits for enterprises, it will not only further reduce the carbon sink cost, but also attract more enterprises participating in NAP construction.

### 4.2. Sensitive Analysis of Wood Market Price

**Figure 5:**
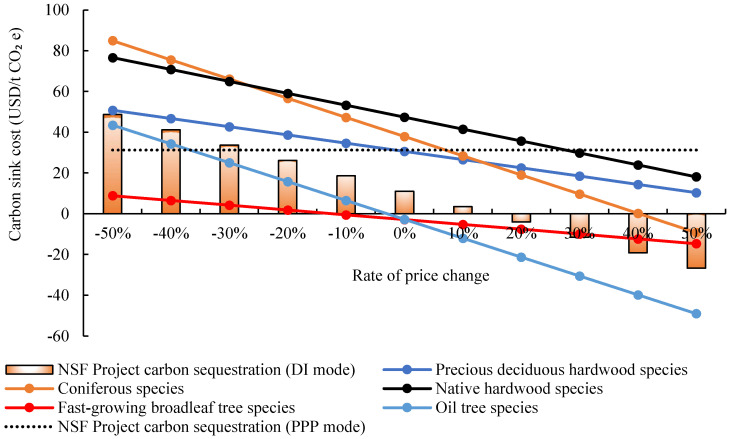
Carbon sink cost dynamic with wood market price change in Laohekou NAP.

The above results demonstrate that using DI investment mode in the Laohekou NAP has better returns, and carbon sink production is feasible only by considering the timber benefits. The benefits of NAP are directly associated with timber prices. From Formulas (14) and (15), the wood price only affects the carbon sink cost under the DI mode, because the timber will not eventually be cut down and sold by the enterprise under the PPP mode. Thus, this section focuses on the wood price influence on carbon sink cost under DI mode. The carbon sink cost of the five tree species and 20-year NAP have been calculated when the wood price changes in the range of [−50%, 50%] (Figure 5). It can be found that the cost of carbon sinks is negatively correlated with the wood price, indicating that the increase in the wood price will lead to a linear decrease in the carbon sink cost under DI mode. Besides, the internal and external factors of wood price formulation, such as the cost of the product, the market demand, buyers, and government, will affect the carbon sink cost under DI mode. When the wood price drops by −26.82%, the carbon sink cost of the DI mode is equal to the PPP mode, and then gradually lower than the PPP. Such information shows that the DI mode is more feasible than the PPP mode only when the wood price is not lower than 73.18% of the current price.

For different tree species under DI mode, Coniferous species and Oil tree species are the most sensitive to changes in wood prices among five tree species, followed by Native hardwood species and Precious deciduous hardwood species, and fast-growing broadleaf tree species is the weakest. Therefore, project implementation enterprises under the DI mode can implement some financial measures (such as hedging) for tree species that are greatly affected by prices to avoid the risk of rising carbon sink cost caused by falling wood prices.

### 4.3. Sensitive Analysis of Wood Yield Rate

**Figure 6:**
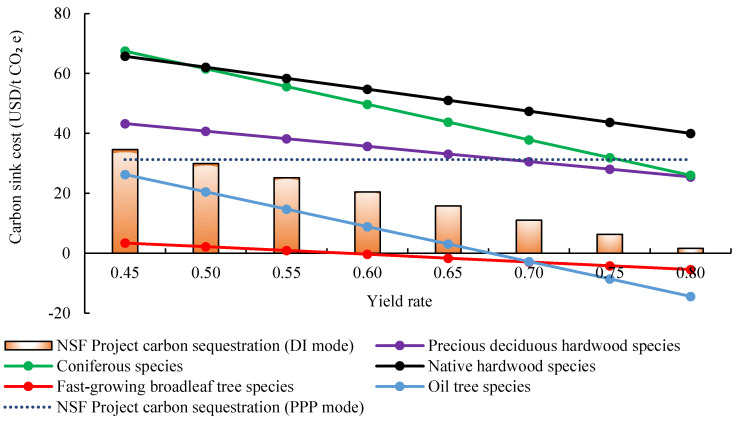
Carbon sink cost dynamic with yield rate in Laohekou NAP.

The yield rate of wood reflects the technical level of wood cutting and utilization. The greater the value, the higher the technical level of cutting and utilization. The yield rate mainly affects the benefit from wood sell, so the change of yield rate will only have impacts on the carbon sink cost in DI mode. This study calculates the changes of different tree species and total carbon sink cost in the 20-year NAP under the DI mode when the yield rate increases from 0.45 to 0.8, which has been shown in Figure 6. It can be found from Figure 6 that the yield rate is linearly negatively correlated with the carbon sink cost. The increase in the yield rate means the increase of the benefit from the wood sell, which reduce the cost of carbon sinks. Furthermore, when the yield rate drops by 0.485, the carbon sink cost of the DI mode is equal to the PPP mode, and then gradually lower than the PPP. That is, PPP is a better investment mode for Laohekou NAPs compared with the DI mode, with the yield rate lower than 0.485.

For different tree species under DI mode, same sensitivity characteristics can be found as the price changes. The main reason is that both the yield rate and price affect the income of wood sales. Besides, this information shows the impact of improved wood yield rate techniques on carbon sink cost for different tree species, which will support enterprises to determine whether to upgrade the cutting technology.

### 4.4. Sensitive Analysis of Discount Rate

**Figure 7:**
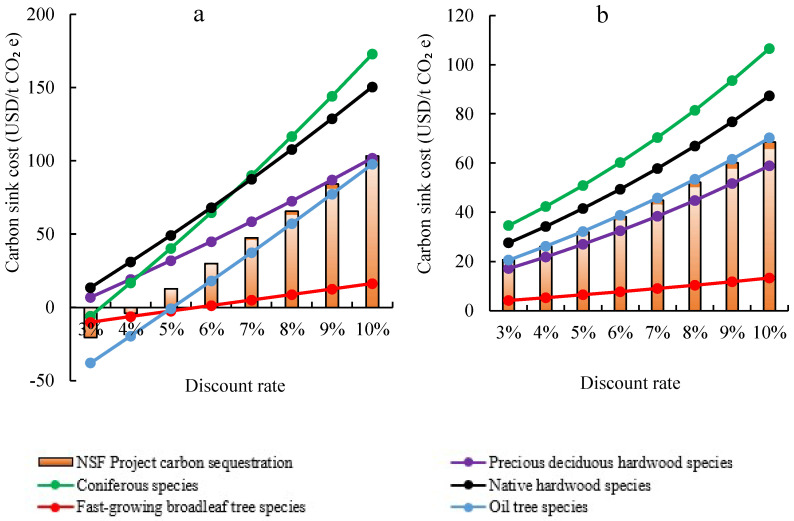
Carbon sink cost dynamic with discount rate change under DI (**a**) and PPP (**b**) investment mode in Laohekou NAP.

The discount rate reflects the time cost of capital. The greater the value, the higher the carbon sink cost. The changes in carbon sink cost under the DI (Figure 7a) and the PPP (Figure 7b) investment mode have been calculated when the discount rate increases from 3% to 10%. It is noteworthy that the carbon sink cost under the DI mode is more sensitive than the PPP mode. The carbon sink cost of DI mode varies in the range of [−20.44, 103.29] USD/t CO_2_e, which is much larger than the [20.75, 68.56] USD/t CO_2_e under PPP mode. That is, changes in the market discount rate have a greater impact on the DI than PPP mode. The main reason for this result is that the government subsidy for the discount rate under the PPP mode certainly reduces the impact of the discount rate on the project cost. Besides, it is noteworthy that when the discount rate is higher than 6.77%, the carbon sink cost under PPP mode will be lower than the DI mode. To reduce the carbon sink cost, PPP is a better option at a high discount rate.

According to the above results, the PPP mode is more capable of resisting changes in the discount rate of the financial market, and the PPP mode can provide stronger robustness and lower risks for enterprises. When making a tradeoff between DI and PPP investment mode, enterprises should take the discount rate into consideration to reduce the cost of carbon sink as much as possible. The sensitivity in DI mode and PPP mode is ranked from high to low as Coniferous species, Native hardwood species, Oil tree species, Precious deciduous hardwood species, and fast-growing broadleaf tree species, which is the opposite of the ranking of carbon storage per unit area of the tree species. Such results indicate that the stronger the carbon sink capacity of the tree species, the less it will be affected by changes in the discount rate, which can help relevant enterprises to screen suitable tree species in future NAPs to resist the risks arising from market interest rate fluctuations.

## 5. Discussion

The opportunity cost of land use and the carbon release cost of wood products were considered in the forestry carbon sink cost calculation under the PPP and the DI investment mode in this study. The case study illustrated that such work was a meaningful attempt at a decision-making tool to help determine the investment mode of an afforestation project. Besides, through the proposed model, the difference of government subsidies between PPP and DI investment model can be measured and compared, which is rarely found in previous studies. However, although multiple factors have impacts on carbon sink cost formulation, we can hardly integrate all of them in one model to calculate the cost of carbon sink. The proposed model in this study took the major factors affecting the carbon sink cost into consideration to attempt to estimate the possible dynamic change of carbon sink cost.

The proposed model was applied to the afforestation project in Laohekou City, which is one of China’s national afforestation projects. The obtained results of the carbon sink costs are within the range (3~280 USD/t) of global forest [29], indicating that the research results are reasonable and reliable. According to the multiple province research from Zhong et al. [30], the average carbon sink cost of afforestation project in China is 171.88 USD/t, and the carbon sink cost in Hubei province is 193.27 USD/t, which is relatively higher than the results of this study. Such a difference is mainly caused by the following reasons: (1) they only took DI mode into account; (2) they used net primary production (NPP) for estimating the carbon sink of forest; (3) they ignored the different characteristics of various tree species of the afforestation project. Although the results of their research have a certain reference significance, they can hardly help to identify the occasions that different tree species and investment mode applied to reduce the cost. The current market price of carbon sink is 2.09–13.43 USD/t [6], which is much lower than calculated carbon sink costs in most cases, especially for PPP mode. This shows that PPP mode cannot effectively reduce carbon sink costs and attract enterprise investment at present in constructing NAPs of China. Therefore, from the perspective of promoting the development of China’s afforestation projects, there is still room for improving subsidy in PPP for constructing NAPs in the future.

Although obtained results can provide decision support for NAPs construction, there are some limitations in this study. Firstly, this study ignored the costs of information for buyers and sellers of forest carbon sinks, and the determination of the buyer and seller costs of transaction contracts, risk management costs, etc., which may have negative impacts on the cost estimation of carbon sinks afforestation projects. Secondly, as a prerequisite for the model operation, massive basic data collection and field investigations must be carried out in the early stage. The lack of necessary data and investigations would limit the application of the presented approach in other similar regions. Thirdly, the tree species were divided as independent five types in this study, but these tree species are often mixed in actual afforestation projects, which may increase the difficulty of area calculation in modeling. Fourthly, the timber output rate, wood density, biomass expansion factor, and above-ground biomass ratio are assumed to be constant in this study. With the cutting and renewal of wood, the above parameters may change due to changes in land productivity. These are issues to be further studied in the future. Besides, the specific measures of supporting NAPs in China was considered in this study. If the proposed carbon sink cost model will be applied to other cases, it can be modified according to the practical regional policies. Thus, these parts should be integrated when developing a new approach to help reduce limitations and improve the applicability and portability of the model.

## 6. Conclusions

Based on the afforestation cost method, this study constructed a forestry carbon sink cost model considering the different impacts of DI and PPP investment modes on the calculation of carbon sink cost. The proposed model was applied to a 20-year national afforestation project of Laohekou City, Hubei Province in China to identify the advantages of two investment modes and impacts of driven factors on carbon sink cost. After analyzing rotation period, wood market price, wood yield rate, and discount rate in detail, the following conclusions were generated.

(1) With the increasing of rotation period, wood market price, and yield rate of timber, the carbon sink cost of Laohekou NAP gradually decreases. The discount rate has the opposite dynamic trend with carbon sink cost. The PPP mode is more feasible than the DI mode only when the wood price is lower than 73.18% of the current price, the yield rate is lower than 0.485, and the discount rate is higher than 6.77%.
(2) DI mode is a better option than PPP mode to reduce the carbon cost of Laohekou NAP in the current situation. Existing subsidy measures are not enough to motivate enterprises to choose the PPP mode. If the local government wants to attract business participation in PPP, existing subsidy measures are not enough. If the government wants to implement the PPP mode in Laohekou NAP, some cost reduction measures, such as extending the benefit period of enterprises and increasing subsidies, should be considered.
(3) Tree species have different characteristics in growth rate, carbon sink capacity, market price, etc. When determining tree species for NAP construction, these factors should be synthetically considered to make the tradeoff between economic benefit and risk, which will support best management practice.

This study focuses on the carbon sink cost of a specific NAPs. Although the results obtained have certain reference value, there are some limitations in the study. For example, there are many factors that affect the cost of carbon sink, such as ground conditions, planting structure, management methods, and regional policies. These will be future works in this field.

## Figures and Tables

**Table 1:** The detailed data of five tree species in Laohekou NAP required by the model.

Items	Unit	Precious Deciduous Hardwood Species	Coniferous Species	Native Hardwood Species	Fast-Growing Broadleaf Tree Species	Oil Tree Species
Planting area	ha	200.00	333.33	286.67	333.33	1346.67
Planting density	Plants/ha	1167	1111	1000	625	1667
Market price of wood	USD/m^3^	187.63	187.63	187.63	90.06	187.63
Planting cost	USD/ha	7769.00	12,123.43	10,009.99	6744.32	6706.59
Raising cost	USD/ha	3782.52	7204.80	5403.60	4728.15	5403.60
Transport cost	USD/m^3^	22.52	22.52	22.52	22.52	22.52
Wood density	kg/m^3^	0.60	0.28	0.44	0.44	0.24
Biomass expansion factor		1.67	1.51	1.59	1.83	1.93
The average carbon content		0.50	0.50	0.48	0.47	0.47
Root biomass ratio		0.26	0.32	0.29	0.25	0.27

## Data Availability

Not applicable.
